# Perineal Groove: Is It More Common Than We Think? Clinical Characteristics of Four Cases and Review of Literature

**DOI:** 10.3390/pediatric13030056

**Published:** 2021-08-09

**Authors:** Hussein Naji, Rola Ali Hassan

**Affiliations:** 1 Department of Pediatric Surgery, Mediclinic Parkview Hospital, Dubai 51122, United Arab Emirates; 2 College of Medicine, Mohammed Bin Rashid University of Medicine and Health Sciences, Dubai 505055, United Arab Emirates; ria050@hotmail.com

**Keywords:** perineal groove, anal fissure, children, anorectal malformation

## Abstract

**Background**: Perineal groove is a very rare congenital malformation that usually occurs in females. It has been described as a wet, un-epithelialized mucus tissue extending from the posterior vaginal fourchette to the anterior anal opening. It is generally asymptomatic and self-limited. Due to its rarity, it is often unfamiliar to clinicians, often leading to a missed diagnosis or unnecessary interventions. **Methods**: During the period from September 2017 to September 2020, four patients (two newborns and 2 infants) were diagnosed with perineal grooves. They were referred to the pediatric surgery clinic because of abnormal findings during their genital examinations. During the same period of time, the clinic received 12 other new patients with various forms of anorectal malformations. **Results**: All four patients were girls. During examinations by their respective pediatricians, an abnormality in the perineum was noted in each of the patients. The depth of the grooves varied between the four patients and extended from the anus to the vaginal fourchette. None of the patients experienced any other symptoms related to the malformation. All the cases were referred to the pediatric surgeon by their pediatricians as a diagnosed anal fissure or abnormal finding in the genitalia. In all four patients, the mothers did not take folic acid during the pregnancy. The four patients were observed, and no surgery was needed; in three of the cases, there was a complete spontaneous resolution while the fourth patient still remains under observation. **Conclusions**: Perineal groove is a rare malformation with a low incidence rate. It is more common in female infants and usually self resolves before the age of 2 years. The condition is diagnosed on clinical examination; however, it is easily misdiagnosed and may lead to unnecessary interventions and surgery.

## 1. Introduction

Perineal groove is a very rare congenital malformation that usually occurs in females. It is part of a broader category of malformations; the incidence rate of anogenital malformations remains underestimated. It has been described as a wet, un-epithelialized mucus tissue that extends from the posterior vaginal fourchette to the anterior anal opening. It is generally asymptomatic and self-limited. Due to its rarity, perineal groove is often unfamiliar to clinicians and can in turn lead to a missed diagnosis and even unnecessary interventions. The causes behind the malformation remain unclear, and few cases have so far been reported. In this study, we aim to highlight this problem by reviewing the literature and exploring the four cases of perineal groove diagnosis in the pediatric surgery clinic from September 2017 to September 2020.

## 2. Case Presentation and Management

During the period from September 2017 to September 2020, four patients (two newborns and 2 infants) were diagnosed with perineal grooves. They were referred to the pediatric surgery clinic because of abnormal findings during their genital examination. During the same period, the clinic received 12 other new patients with various forms of anorectal malformations.

**Figure 1:**
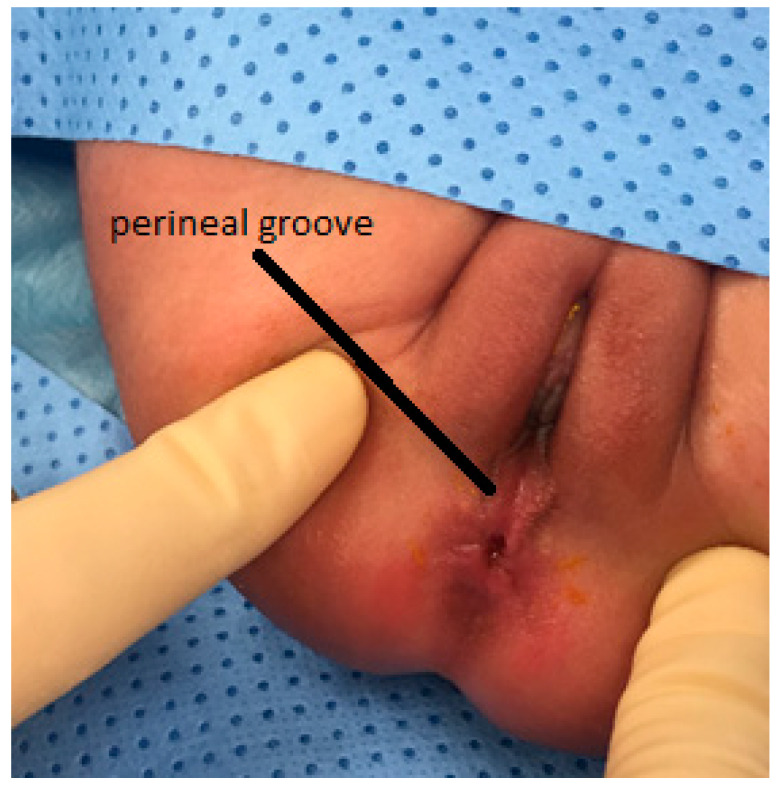
Perineal groove in a 7-day old patient.

**Figure 2:**
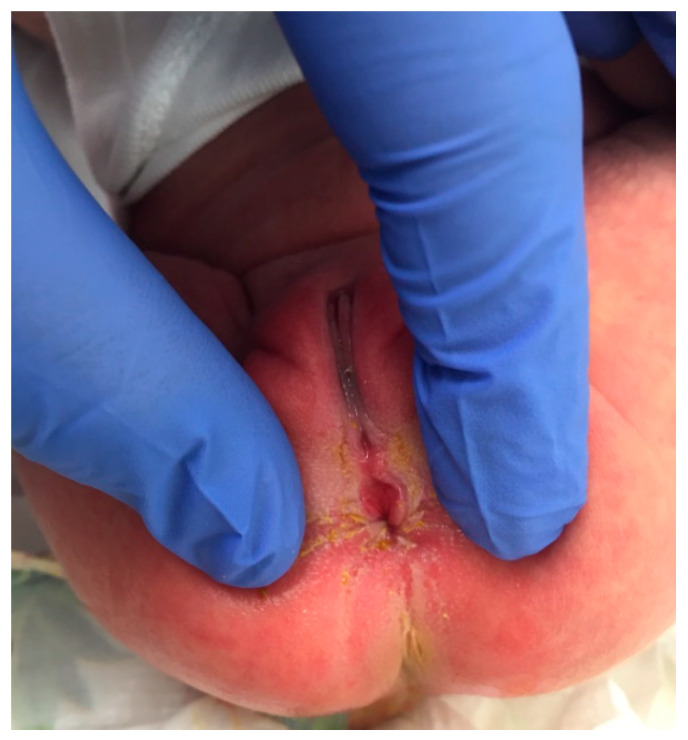
Perineal groove in a 15-day old patient.

All the four patients were girls (Table 1) and an abnormality in the perineum was noted in each of them during examination by their respective pediatricians. All patients had normal urethral and vaginal orifices as well as normal anuses. The depth of the grooves varied between the four patients and extended from the anus to the vaginal fourchette (Figure 1 and Figure 2). None of the patients had any other symptoms related to the malformation. All of the cases were referred to the pediatric surgeon by their pediatricians as an anal fissure or abnormal finding in the genitalia. In all four patients, the mothers did not take folic acid during the pregnancy.

The four patients were observed, and no surgery was needed. Three of the patients experienced a complete spontaneous resolution while the fourth is still under observation.

### 2.1. Case 1

A 7-day old female patient was referred by her neonatologist with a suspicion of a congenital anal fissure. During examination we found a perineal groove. She was born to a 26-year-old gravida 1 para 1 woman who did not take folic acid during her pregnancy. The patient was not noted to have any other associated anomalies during her exam.

She was followed up regularly for 2 years, and it was noted that the perineal groove spontaneously resolved.

### 2.2. Case 2

A 15-day old female also with no other associated medical issues was referred by her neonatologist with an abnormality of the anogenital area. We diagnosed the patient with perineal grove in our clinic. She was born to a 38-year-old woman gravida 3 para 2. The mother did not take folic acid during her pregnancy.

We conducted follow-ups for 18 months, and it appeared that the groove underwent a spontaneous resolution.

### 2.3. Case 3

A 2-month-old female patient presented with a perineal groove. The patient was already known to have a mild ventricular septal defect (VSD). She was born to a 40-year-old mother gravida 2 para 2. The mother did not take folic acid during her pregnancy.

We conducted follow-ups for 18 months. The VSD naturally closed at 9 months of age. The perineal groove also spontaneously resolved at 16 months of age.

### 2.4. Case 4

A 6-month-old female was referred to the clinic by her pediatrician due to abnormal findings in the genitalia.

A perineal groove was found during examination. She was born to a 31-year-old woman gravida 3 para 2 who had not taken folic acid during her pregnancy.

The patient had been observed for 6 months. The perineal groove has not yet been resolved; the patient remains under observation, though there is no plan for surgery at this point.

## 3. Discussion

A perineal groove is a rare entity of anorectal malformations that occurs nearly exclusively in female patients, with only two reported cases in male patients [1,2]. There are no more than 30 publications on this subject and most of these were case presentations [3].

Perineal groove has been described as a wet red sulcus extending from the posterior vaginal fourchette to the anterior anal opening. Usually, it is an isolated finding, but it may be associated with other urological or genital defects such as imperforate anus, perineal ectopic anus, hypospadias or rectal prolapse [2].

The occurrence of perineal groove in male infants points towards its development during the embryological stage. There are at least four different theories regarding its development: (1) failure in the developmental stage of the external genitalia, (2) abnormal fusion of the median genital fold, (3) remnant of the cloacal duct due to a defect in the urorectal septum [4,5,6], (4) a theory related to genetics [3]. In all of these theories, it is noted that the perineal groove is not associated with any abdominal or pelvic anomalies [6].

The types of the perineal grooves have been divided into complete and incomplete. The complete perineal groove extends from the vaginal fourchette to the anus, while the incomplete type either extends from the vaginal fourchette to the middle of the perineum or from the anus to the middle of the perineum [3].

Three criteria need to be met in order to define this malformation: (1) wet groove in the perineum; (2) normal anatomy of the vestibular organs: both vagina and urethra; (3) hypertrophy of minoral tails [3,6,7].

Patients with perineal grooves are generally asymptomatic [5]; however, they might be at increased risk of urinary infections and urinary incontinence or inflammation of the perineal skin [4]. Failure to diagnose this malformation may lead to misdiagnoses, such as inflammation of the area, lichen sclerosis, diaper rash or even sexual abuse [4,5]. Furthermore, it can sometimes be confused with irritant dermatitis, a common cause of diaper rash [8].

Perineal groove is a clinical diagnosis, with a biopsy only rarely performed. When conducted, the histological findings of the biopsy specimens exhibit non-keratinizing squamous epithelium with an intervening area of rectal type or transitional epithelium [2]. While one can perform imaging to screen for any associated regional anomalies, it is a rare finding with very little literature to prove an association. An example is the association of perineal groove with hypospadias and bifid scrotum [2,9].

It is important to properly diagnose perineal groove as the main treatment is conservative observation [1]. Within a year of presentation, a perineal groove has the potential to spontaneously resolve. Surgical excision of the groove might be considered after 2 years of age if the lesion has not re-epithelialized or complications such as recurrent urinary tract infections occur [8,9,10]. Surgical excision is usually indicated for cosmesis or correction of other anomalies such as hypospadias [2,9].

Perineal grooves are noted as anogenital malformations [11]. Histological resection of the area resembled an anorectal transition zone epithelium, implying that the defect occurred during the development of the urorectal septum [4,10].

The benefits of folic acid during early pregnancy in the prevention of neural tube defects is well documented [12,13]. It is still uncertain whether this is true for other congenital birth defects. A single study from China showed a reduced risk of an imperforate anus when a mother took folic acid during pregnancy [12]. We have tried to find an association between folic acid intake and development of perineal groove in our study. The mothers of each of the 4 patients did not take folic acid during pregnancy. However, the study population is extremely small and therefore we cannot draw a definite conclusion. In future, we intend to formulate a multicenter study to look for this association.

## 4. Conclusions

Perineal groove is a rare malformation with a low incidence rate. It predominantly affects female infants and usually self-resolves before the age of 2 years. The condition is diagnosed on clinical examination; however, it is easily misdiagnosed and can thus lead to unnecessary interventions and surgery.

Further studies are needed to identify the relationship between a mother’s folic acid intake and a reduction in perineal groove incidences

## Figures and Tables

**Table 1:** Demographic data of 4 cases.

Case	Age of Patient	Gender	Other Associated Anomalies	Folic Acid Intake during Pregnancy	Mother’s Age and Parity	Follow Up	Outcome
One	7 days	Female	None	None	26 yo G1P1	2 years	Spontaneous resolution
Two	15 days	Female	None	None	38 yo G3P3	18 months	Spontaneous resolution
Three	2 months	Female	Mild VSD closed spontan-eously at 9 months of age	None	40 yo G2P2	18 months	Spontaneous resolution
Four	6 months	Female	None	None	31 yo G3P2	6 months	Under observation

VSD: ventricular septal defect; G: gravida; P: para; yo: years old.

## Data Availability

Not applicable.
